# Immune profiles according to EGFR mutant subtypes and correlation with PD-1/PD-L1 inhibitor therapies in lung adenocarcinoma

**DOI:** 10.3389/fimmu.2023.1137880

**Published:** 2023-03-24

**Authors:** Young Wha Koh, Bumhee Park, Se Hee Jung, Jae-Ho Han, Seokjin Haam, Hyun Woo Lee

**Affiliations:** ^1^ Department of Pathology, Ajou University School of Medicine, Suwon-si, Republic of Korea; ^2^ Department of Biomedical Informatics, Ajou University School of Medicine, Suwon-si, Republic of Korea; ^3^ Office of Biostatistics, Medical Research Collaborating Center, Ajou Research Institute for Innovative Medicine, Ajou University Medical Center, Suwon-si, Republic of Korea; ^4^ Department of Thoracic and Cardiovascular Surgery, Ajou University School of Medicine, Suwon-si, Republic of Korea; ^5^ Department of Hematology-Oncology, Ajou University School of Medicine, Suwon-si, Republic of Korea

**Keywords:** lung adenocarcinoma, EGFR, PD-L1, PD-1, CD8, CD4

## Abstract

**Background:**

We examined the distributions of 22 immune cell types and the responses to PD-1/PD-L1 inhibitors according to EGFR mutation profile, in three independent datasets of lung adenocarcinoma (LUAD).

**Methods:**

We used CIBERSORTx to analyze the distributions of immune cells, and tumor immune dysfunction and exclusion (TIDE) or tumor mutation burden (TMB) to analyze responses to anti-PD-1/PD-L1 therapy, in two public LUAD datasets. The results were verified with a validation set that included patients treated with PD-1/PD-L1 inhibitors.

**Results:**

Compared to EGFR mutants, EGFR wild-type carcinomas had higher numbers of CD8+ T cells, CD4 memory activated T cells and neutrophils, and lower numbers of resting dendritic cells and resting mast cells, in two of the datasets. In our subgroup analyses, CD8+ T cells and CD4 memory activated T cells were more numerous in EGFR rare variants than in wild-types, L858R mutants, and exon 19 deletion mutants. In our TIDE or TMB analyses, EGFR rare variants were predicted to respond better to PD-1/PD-L1 inhibitors than wild-types, L858R mutants, and exon 19 deletion mutants. In the validation set verified by immunohistochemical staining, levels of CD8+ T cells in the EGFR rare variant or wild-type groups were significantly higher than in the EGFR L858R and exon 19 deletion groups. In patients treated with PD-1/PD-L1 inhibitors, the survival rates of patients with EGFR wild-type and rare mutant carcinomas were higher than those with L858R and exon 19 deletion carcinomas.

**Conclusion:**

The EGFR rare mutation form of LUAD shows a higher immune activation state compared to wild-type, L858R, and exon 19 deletion variants, indicating it as a potential target for PD-1/PD-L1 inhibitor therapy.

## Introduction

Among the adenocarcinomas associated with non-smokers in East Asia, EGFR mutations are the most common driver genes, accounting for approximately 60-78% of driver genes in the group (1). After receiving anti-programmed cell death protein 1/programmed death-ligand 1 (PD-1/PD-L1) treatment, adenocarcinoma patients positive for EGFR mutant show poorer responses than those with the wild-type (2). Because many patients in East Asia have EGFR mutations, they are excluded from treatment with PD-1/PD-L1 inhibitors. NSCLC with mutated EGFR has lower tumor mutation burden (TMB) levels than the wild-type, which may affect PD-1 inhibitor treatment (3). A negative correlation has been found between EGFR mutation and PD-L1 expression (3). Patients with EGFR-mutated NSCLC lack T-cell infiltration and have decreased ratios of PD-L1+/CD8+ tumor-infiltrating T cells (3). Single-cell analysis has reported that CD8+ tissue-resident memory (TRM) cells are deficient in EGFR-mutant forms of LUAD, compared to wild-type forms (4). There are many immune cells other than T cells in the tumor microenvironment that can affect anti-PD-1/PD-L1 treatment, but their effects are poorly understood. Studies of the effects of EGFR mutations in patients receiving anti-PD-1/PD-L1 therapy are rare.

CIBERSORTx is an analytical tool that uses gene expression data to evaluate cell type abundance (5). Tumor immune dysfunction and exclusion (TIDE) is a machine learning tool that uses gene expression data to evaluate T cell dysfunction and exclusion, and to predict tumor responses to anti-PD-1/PD-L1 therapy (6). In this study, we investigated the distributions of 22 immune cells according to the presence or absence of EGFR mutations using two public LUAD gene expression datasets and the CIBERSORTx tool. The response rates to anti-PD-L1/PD-1 treatment according to the presence of EGFR mutation were verified using the TIDE tool or tumor mutation burden (TMB). We also analyzed whether the response varied depending on the presence of EGFR mutation and immune cell type in patients who received anti-PD-L1/PD-1 treatment. Lastly, we investigated differences in the distributions of immune cells and TIDE scores, according to EGFR mutation subtype.

## Materials and methods

### Study population and EGFR test

Two public gene expression data sets (510 and 110 samples) and one validated data set (203 samples) were studied. We extracted two LUAD mRNA datasets from cBioportal databases (http://cbioportal.org) (7). The first dataset comprised 510 samples (pancancer dataset, wild-type: 444, L858R: 22, exon 19 deletion: 25, rare: 19) (8) and the second dataset (cptac dataset, wild-type: 72, L858R: 16, exon 19 deletion: 16, rare: 6) comprised 110 samples (9). The rare mutations in the first data set consisted of two exon 20 insertions, three G719X mutations, and 14 other mutations. The rare mutations in the second data set consisted of four G719X mutations and two other mutations. We were able to identify EGFR mutation profiles in all datasets. We obtained TMB scores from the cBioportal databases for each case. The demographic and clinical characteristics of validation set are summarized in **Table 1**. A total of 203 patients were enrolled (wild-type: 84, L858R: 36, exon 19 deletion: 46, rare: 37), 49 were treated with PD-1/PD-L1 inhibitors (wild-type: 31, L858R: 7, exon 19 deletion: 8, rare: 3) and 154 were not (wild-type: 53, L858R: 29, exon 19 deletion: 38, rare: 34). The rare mutations in the treated group consisted of one exon 20 insertion and two G719X mutations, and the rare mutations in the non-treated group consisted of 16 exon 20 insertions, 12 G719X mutations and six other mutations. Ethical approval was granted by the Institutional Review Board of Ajou University School of Medicine (AJOUIRB-KSP-2020-396 and 2020-12-28).

**Table 1:** Demographic and clinical characteristics of patients.

Variable	Number (%)
Age, median (range) (years)	64 (35–85)
Male sex	124 (61.1%)
TNM 8th edition	
Stage I	71 (35%)
Stage II	24 (11.8%)
Stage III	55 (27.1%)
Stage IV	53 (26.1%)
EGFR test method	
Real-time PCR	166 (81.8%)
Next-generation sequencing	37 (18.2%)
EGFR results	
Wild	84 (41.4%)
L858R	36 (17.7%)
Exon 19 deletion	46 (22.7%)
Rare	37 (18.2%)
Smoking history	
Presence	96 (59.6%)
Absence	65 (40.4%)
PD-L1/PD-1 inhibitor	
Treatment	49 (24.1%)
No treatment	154 (75.9%)

Smoking history was obtained in 161 patients.

### Immunohistochemistry of CD8

Immunochemical staining was performed for surgical resection samples using a tissue microarray, and biopsy samples were performed for whole sections. Anti-CD8 antibodies (clone C8/144B, DAKO) were used in analyses. For evaluation of CD8 immunostaining, membrane-positive cells were measured at three locations and the average value was calculated.

### CIBERSORTx and TIDE

We used the CIBERSORTx tool to identify 22 human immune cell subpopulations in lung adenocarcinoma samples (5). We used the TIDE tool to identify four biomarkers: TIDE, interferon gamma gene signature, T-cell-inflamed signature, and PD-L1 (6).

### Statistical analyses

We used Spearman's rank coefficient or Kruskal–Wallis H test as nonparametric measures of rank correlation. Pearson's chi-squared test was used for statistical tests on categorical data. Survival analysis was performed using a Kaplan–Meier estimator. IBM SPSS Statistics for Windows, version 25.0 (IGM Inc., Armonk, NY, USA) or R version 3.5.3 (http://www.r-project.org/) were used for all analyses. All *p* values less than 0.05 were considered statistically significant.

## Results

### Differences in 22 immune cell components according to EGFR mutation profiles

**Figure 1:**
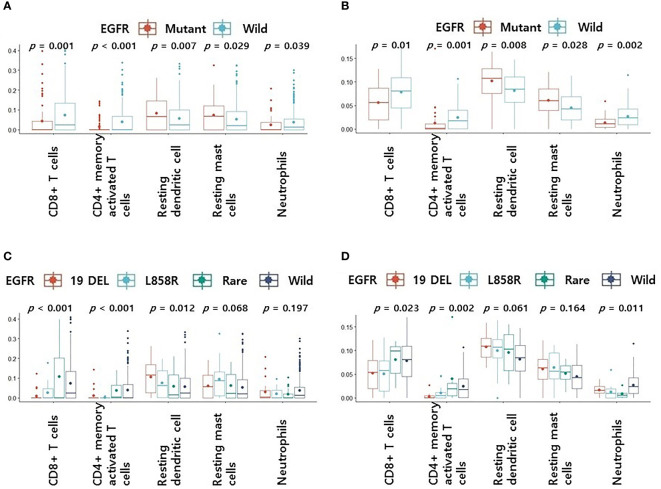
Differences in 5 immune cell components according to EGFR mutation profiles. **(A)** Changes in levels of 5 immune cell components according to EGFR mutations in pancancer dataset **(A)** and cptac dataset **(B)**. Changes in levels of CD8+ T cells, CD4 memory activated T cells, resting dendritic cells, resting mast cells and neutrophils according to EGFR mutational subtypes in pancancer dataset **(C)** and cptac dataset **(D)**. The small dot in the boxplot is the mean value. 19 DEL, exon 19 deletion.

We confirmed differences in 22 immune cell components according to EGFR mutation profiles in two public LUAD datasets. In the pancancer dataset, CD8+ T cells (*p* = 0.001), CD4 memory activated T cells (*p* < 0.001), follicular helper T cells (*p* = 0.012), resting NK cells (*p* = 0.037), and neutrophils (*p* = 0.039) were significantly more abundant in the EGFR wild-type group than in the mutation group. However, CD4 naive T cells (*p* = 0.009), resting dendritic cells (*p* = 0.007), activated dendritic cells (*p* = 0.027), and resting mast cells (*p* = 0.029) were significantly less abundant in the EGFR wild-type group than in the mutation group. In the cptac dataset, naïve B cells (*p* = 0.036), plasma cells (*p* = 0.003), CD8+ T cells (*p* = 0.01), CD4 memory activated T cells (*p* = 0.001), and neutrophils (*p* = 0.002) were significantly more abundant in the EGFR wild-type group than in the mutation group. However, CD4 memory resting T cells (*p* = 0.01), monocytes (*p* = 0.015), M2 macrophages (*p* = 0.048), resting dendritic cells (*p* = 0.008), and resting mast cells (*p* = 0.028) were significantly less abundant in the EGFR wild-type group than in the mutation group. Some common results found between the two datasets were higher levels of CD8+ T cells, CD4 memory activated T cells and neutrophils, and lower levels of resting dendritic cells and resting mast cells in the EGFR wild-type groups versus the mutation groups (**Figure 1**).

We then performed subgroup analyses according to EGFR mutation subtype for four groups: wild-type, L858R, exon 19 deletion, and rare mutation. Other than L858R and exon 19 deletion, all mutations were classified as rare. Levels of CD8+ T cells, CD4 memory activated T cells, resting dendritic cells, resting mast cells, and neutrophils, which showed significant differences between the two datasets, were included in our subgroup analyses.

In the pancancer dataset, the rare variant had the highest CD8+ T cell and CD4 memory activated T cell levels among the four groups (*p* < 0.001, **Figure 1C**). Levels of CD8+ T cells and CD4 memory activated T cells were higher in the rare mutant and wild type than in the exon 19 deletion and L858R (*p* < 0.001, **Figure 1C**). There were no differences in resting dendritic cells, resting mast cells, and neutrophils levels in rare variant, exon 19 deletion, and L858R groups (**Figure 1C**). In the cptac dataset, the rare variant group also had the highest CD8+ T cell and CD4 memory activated T cell levels among the four (**Figure 1D**). Levels of CD8+ T cells or CD4 memory activated T cells were also higher in the rare mutant and wild type groups compared to the exon 19 deletion and L858R mutation groups (*p* = 0.023 and *p* = 0.002, respectively, **Figure 1D**). There were also no differences in resting dendritic cell, resting mast cell, and neutrophil levels in the rare variant, exon 19 deletion, and L858R groups (**Figure 1D**).

### Differences in TIDE score or TMB according to EGFR mutation profile

**Figure 2:**
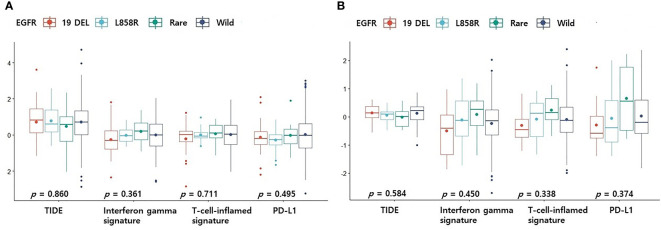
Differences in TIDE-related biomarkers according to EGFR mutational subtypes. Changes in levels of TIDE-related biomarkers according to EGFR mutational subtypes in pancancer dataset **(A)** and cptac dataset **(B)**. The small dot in the boxplot is the mean value. 19 DEL, exon 19 deletion.

**Figure 3:**
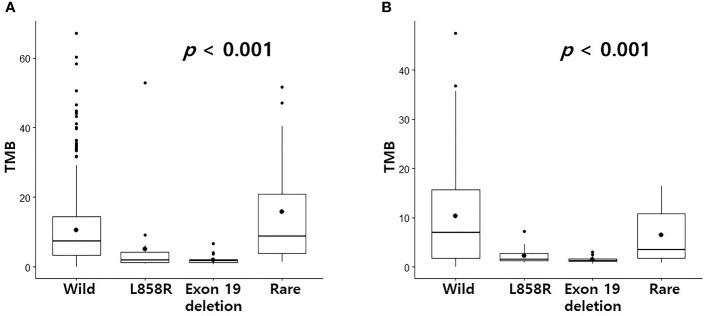
Differences in TMB according to EGFR mutational subtypes. Changes in levels of TMB according to EGFR mutational subtypes in pancancer dataset **(A)** and cptac dataset **(B)**. The small dot in the boxplot is the mean value.

CD8+ T cells or CD4 memory activated T cells are immune cells closely related to immunotherapy (10, 11). Because the levels of CD8+ T cells and CD4 memory activated T cells were surprisingly high in the rare variant group, we investigated whether the TIDE score was different for each EGFR subtype. We verified differences in four TIDE-associated biomarkers according to EGFR subtype. In previous studies, patients with low TIDE (6), high interferon gamma signature (12), high T cell inflamed signature (13) and high PD-L1 (14) responded better to PD-1/PD-L1 inhibitors. In the pancancer dataset, although not statistically significant, the interferon gamma signature and T cell inflamed signature of the rare variant were the highest among the four groups, and the TIDE score was the lowest among the four groups (**Figure 2A**). PD-L1 expression in the rare variant group was the second highest after the wild-type group (**Figure 2A**). In the cptac dataset, although not statistically significant, the interferon gamma signature, T cell inflamed signature, and PD-L1 expression in the rare variant group were also the highest among the four, and the TIDE score was the lowest (**Figure 2B**). In the pancancer dataset, the TMB score of the rare variant group was the highest among the four (*p* < 0.001, **Figure 3A**). In the cptac dataset, the TMB score of the rare variant group was the second highest after the wild-type (*p* < 0.001, **Figure 3B**). The TIDE analysis result was that, of the four group (including the wild-type), the rare variant group was most likely to respond well to PD-L1/PD-1 inhibitor treatment.

### Differences in CD8+ T cells according to EGFR mutation profile in the validation set

**Figure 4:**
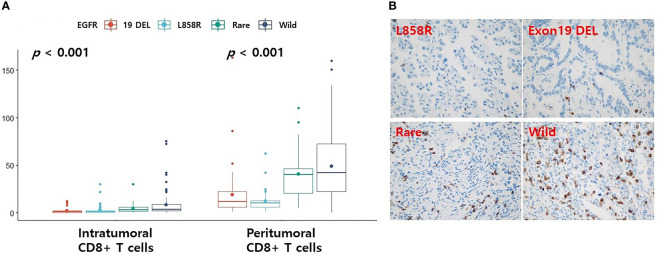
Differences in the levels of CD8 according to EGFR mutational subtypes analyzed by immunohistochemistry. **(A)** Changes in levels of CD8 according to EGFR mutational subtypes. Representative immunohistochemical images of CD8 expression. **(B)** Case with EGFR L858R or exon 19 deletion mutation is associated with low CD8+ T cells. Case with EGFR rare variant or wild-type is associated with high CD8+ T cells. The small dot in the boxplot is the mean value. 19 DEL, exon 19 deletion.

Because we could not find an immunohistochemical antibody that could clearly detect CD4 memory activated T cells, only CD8+ T cells were re-validated by immunohistochemistry. Levels of CD8+ T cells were found to be higher in the EGFR wild-type and rare variants groups than in the L858R and exon 19 deletion groups in both tumor and peritumoral regions (**Figure 4A**, all *p* < 0.001). Representative figures for EGFR wild, L858R, exon 19 deletion, and rare mutation results are summarized in **Figure 4B**.

### Smoking status according to EGFR subtype

**Figure 5:**
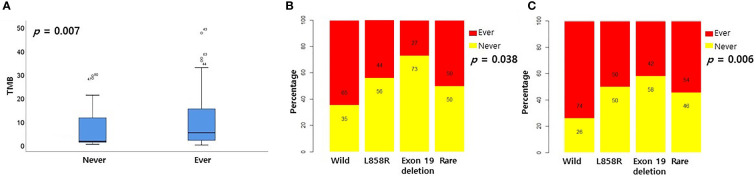
Relationship between smoking history and EGFR mutational subtypes. **(A)** Relationship between smoking history and TMB in cptac dataset. Relationship between smoking history and EGFR mutational subtypes in cptac **(B)** and validation **(C)** dataset.

Previous studies revealed that patients with smoking histories had high TMB levels and responded well to PD-1 inhibitors (15). Therefore, we examined the relationship between smoking history and EGFR subtype. However, since there was no information on smoking history in the pancancer data set, only the cptac and validation datasets were analyzed. In the cptac dataset, the TMB score was significantly higher for those with a history of smoking than those without a history of smoking (**Figure 5A**, *p* = 0.007). Smoking history was most frequent in wild-type patients and least frequent in exon 19 deletion patients. (**Figure 5B**, *p* = 0.038). In the validation dataset, smoking history was also most frequently present in the wild-type group, and least frequent in the exon 19 deletion group (**Figure 5C**, *p* = 0.006).

### Prognostic role of EGFR mutation in patients using PD-L1/PD-1 inhibitors

**Figure 6:**
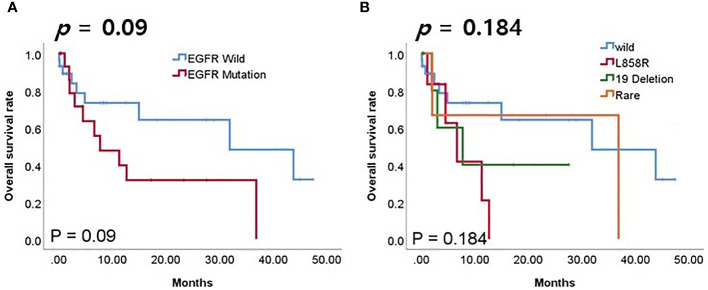
Survival analyses according to EGFR mutation in patients receiving PD-1/PD-L1 inhibitors. **(A)** Overall survival (OS) according to EGFR mutation. **(B)** OS according to EGFR mutation subtype.

We investigated the prognostic role of EGFR mutation in patients using PD-L1/PD-1 inhibitors. Although the difference was not statistically significant, the EGFR mutation group had lower overall survival (OS) rates compared to the wild-type (**Figure 6A**, *p* = 0.09). Although the difference was not statistically significant, groups with EGFR wild type or rare mutations had higher rates of OS compared to groups with L858R or exon 19 deletion mutations (**Figure 6B**, *p* = 0.184).

## Discussion

We found that levels of CD8+ T cells or CD4 memory activated T cells were higher in EGFR wild-type and rare variant cancers than in EGFR L858R and exon 19 deletion types. Among patients using PD-1/PD-L1 inhibitors, those with EGFR wild-type and EGFR rare mutations had better prognoses than those with EGFR L858R and exon 19 deletion mutations. CD8+ T cells are the most potent effectors in the anti-cancer immune response, and serve as the backbone of cancer immunotherapy (11). Immune checkpoint inhibitors block inhibitory immune receptors and aim to activate dysfunctional CD8+ T cells (11). Immune cold tumor is a common immunotherapy-resistant phenotype observed in solid tumors (16). The definition of hot and cold tumors depends in part on the extent and location of infiltrating CD8+ T cells (17). Therefore, it is predictable that hot tumors respond well to immunotherapy and cold tumors do not. One previous study also reported that EGFR-mutated NSCLC carcinomas were free of T cell infiltration and had decreased proportions of PD-L1+/CD8+ tumor-infiltrating T cells (3). Studies of patients using PD-1/PD-L1 inhibitors have shown that NSCLCs carrying EGFR mutations are associated with poor responses, suggesting that these mutations are associated with a smaller proportion of CD8+ T cells (18). Another study showed that lung cancer patients with the L858R EGFR mutation had more inflammatory tumors with higher CD4 and CD8+ T cell expressions compared to those with the exon 19 deletion mutation (19). However, we found no significant differences in CD4 and CD8+ T cells between L858R and exon 19 deletion groups. Infiltration of CD8+ T cells and neutrophils was observed more frequently in the rare EGFR mutant group than in the L858R and exon 19 deletion groups.

CD4+ T cells have recently been highlighted as playing important roles in regulating the anti-tumor immune response (10). One study found that a higher number of CD62L^low^ CD4+ T cells prior to PD-1 blockade therapy was significantly associated with better responses (20). Laheurte et al. reported that higher levels of anti-TERT Th1^high^ CD4+ T cells in the peripheral blood was correlated with better clinical outcomes in NSCLC patients (21). Activated CD4+ T cells secrete interleukin (IL)-2 to directly activate CD8+ cytotoxic T cells (22). CD4+ T cells can induce antitumor responses by secreting interferon gamma and tumor necrosis factor-α (TNFα) (23). CD4+ T cells also induce humoral responses to tumor antigens on B cells through the interaction of CD40 with CD40 ligands (10). High CD4 memory activated T cells was significantly associated with better overall survival in gastric cancer (24). In head and neck squamous cell carcinoma, the group with high activated CD4(+)CD69(+) T cells had a better prognosis than the group with low CD4(+)CD69(+) T cells (25).

In our study, the five biomarkers used to predict response to PD-L1/PD-1 inhibitors were TIDE, interferon gamma gene signature, T cell inflammatory signature, PD-L1, and TMB. Ayers et al. found that the interferon gamma gene signature could predict responses to PD-1 inhibitors in 220 patients with nine cancers, including NSCLC (12). The T cell inflammatory signature is a well-known indicator of T cell dysfunction (13). PD-L1 expression is the most frequently used biomarker for the use of PD-L1/PD-1 inhibitors in solid cancers, including NSCLC, in clinical practice (26). Therefore, these four biomarkers are currently the most widely used biomarkers for PD-L1/PD-1 inhibitors. Although not statistically significant, rare variants were predicted to respond best to PD-L1/PD-1 inhibitor treatment in four TIDE biomarkers. It is well known from previous studies that tumors with high TMB have more neoantigens and more immunogenicity (27). Rizvi et al. reported that high TMB levels in tumors of NSCLC patients treated with pembrolizumab had good prognoses (27). Although in our study only TMB was statistically significant and the other factors were not, due to the small number of samples of rare variants, PD-L1/PD-1 inhibitor treatment should be considered for the treatment rare variant NSCLC tumors in the future, as they are expected to respond better than wild-type ones.

Negrao et al. reported that EGFR exon 20 mutations were associated with low expression of PD-L1 (28). Therefore, EGFR exon 20 mutations were also predicted to have less benefit from PD-1 inhibitors. Hastings et al. also reported that the exon 20 insertion mutation was associated with low levels of TMB, whereas the G719X mutation was associated with high TMB levels (15). The G719X mutation was also associated with higher expression of TMB and PD-L1 than the classical EGFR mutation in another study of NSCLC patients (29). In the two public datasets we reviewed, the frequency of exon 20 insertion was relatively low and the frequency of G719X mutation was relatively high (12% vs. 18% in the pancancer dataset and 0% vs 66% in the cptac dataset, respectively). In our survival analysis of our validation dataset, the frequency of the G719X mutation was higher than that of exon 20 insertion (66% vs. 33%). In our dataset, the high frequency of the G719X mutation and the low frequency of the exon 20 insertion mutation may have been the causes of high CD8+ T cell scores and high TMBs.

Hastings et al. reported that a smoking history was associated with a high TMB level and responded well to immune checkpoint inhibitors (15). A positive correlation between smoking history and TMB levels was also identified in our cptac data set. In previous report, smoking history was observed more frequently with the L858R mutation than with the exon 19 deletion (15). In our two data sets, smoking history was also found more frequently with the L858R mutation compared to the exon 19 deletion. Among the four EGFR subtypes of our two data sets, smoking history was most common in the wild type and second most common in rare mutations.

Compared to other studies in the past (15, 28, 30), the sample size of our study is relatively too small. Two studies (Hastings et al's cohort (n=554) (15) and Negrao et al's cohort (n=4189) (28))reported that EGFR exon 20 mutations were associated with reduced benefit from PD-1 inhibitors. Mazieres et al. found no difference in survival between rare and classical EGFR mutations on PD-1/PD-L1 treatment in 551 NSCLCs (30). However, experiments with a relatively large number of samples also reported that rare mutations in EGFR were associated with high levels of TMB or PD-L1 expression. In an experiment targeting 1,111 NSCLC patients, it was found that the levels of TMB and PD-L1 in the G719X mutation were higher than those in the classical EGFR mutation (29). In 2417 NSCLC patients, PD-L1 high-expression was more likely to shown with G719X/S768I/exon 20 insertion than with classical EGFR /L861Q mutation (31). In 982 NSCLCs, rare EGFR mutations (G719X, L861Q, S768I, exon 20 insertion) showed statistically significantly higher PD-L1 expression than classical EGFR mutations (32). Although our results indicate that patients with rare EGFR mutations are more likely to respond to PD-L1/PD-1 inhibitors in three independent data sets, the prescription of PD-L1/PD-1 inhibitors for rare EGFR mutations needs to be validated with more samples.

Our study had some limitations. First, although numerous EGFR rare mutations have been reported, these were combined and analyzed together in this study. As a result, the immune profiles associated with specific rare mutations or their relationships to PD-1/PD-L1 inhibitors were not examined. Because the number of rare EGFR mutations was small, it was difficult to perform subgroup analysis for rare EGFR mutations. The immune characteristics of specific rare mutations should be investigated in larger-scale studies. Second, our validation set consisted of 203 patients, of which 49 were treated with PD-1/PD-L1 inhibitors. The small number of patients divided into four groups (EGFR wild, L858R, exon 19 deletion, and rare) for analysis may have limited the interpretation of the results. Third, we did not perform CIBERSORTx, TIDE, and immunohistochemistry analyses on the same LUAD dataset. Although similar results were obtained for all three datasets, our results should be validated using the same dataset. Fourth, we could not confirm the distribution of CD4 memory activated T cells in the validation set. Because the level of CD4 memory activated T cells in the two public datasets was the highest in the rare variant, it is thought that CD4 memory activated T cells may affect immunotherapy.

In this study, we investigated differences in 22 immune cell components following EGFR mutation in 620 LUADs in two public databases, for the first time. Subgroup analysis revealed that the rare variant group had the highest CD8+ T cell and CD4 memory activated T cell levels among the four groups, including the wild-type. TIDE and TMB analyses also showed that rare EGFR variants was more likely to respond to PD-L1/PD-1 inhibitors than wild-type, L858R-mutated, and exon 19 deletion-mutated EGFR lung cancers. A validation set using CD8+ T cell immunochemical staining demonstrated an immune profile similar to the previous two data sets for EGFR rare mutations, and a better prognosis for these cancer types than L858R and exon 19 deletions, with PD-1/PD-L1 inhibitor treatment. The results of this study indicate that rare EGFR mutations may be potential targets for PD-1/PD-L1 inhibitors.

## Data availability statement

The original contributions presented in the study are included in the article/supplementary materials. Further inquiries can be directed to the corresponding author.

## Ethics statement

The studies involving human participants were reviewed and approved by The Institutional Review Board of Ajou University School of Medicine. Written informed consent for participation was not required for this study in accordance with the national legislation and the institutional requirements.

## Author contributions

YK designed the study. SH, J-HH, HL, and YK collected the data, SH, J-HH, HL, BP, SJ, and YK performed the experiments and analyzed the data. SH, J-HH, HL, and YK wrote the manuscript. All authors contributed to the article and approved the submitted version.
